# Extensive Description and Comparison of Human Supra-Gingival Microbiome in Root Caries and Health

**DOI:** 10.1371/journal.pone.0117064

**Published:** 2015-02-06

**Authors:** Lin Chen, Bingcai Qin, Minquan Du, Huanzi Zhong, Qingan Xu, Yuhong Li, Ping Zhang, Mingwen Fan

**Affiliations:** 1 The State Key Laboratory Breeding Base of Basic Science of Stomatology (Hubei-MOST) & Key Laboratory of Oral Microbiology Ministry of Education, School & Hospital of Stomatology, Wuhan University, Wuhan, China; 2 Department of Endodontics, Guangdong Provincial Stomatological Hospital, Southern Medical University, Guangzhou, China; 3 BGI-Shenzhen, Shenzhen, China; 4 Department of Prevention, School & Hospital of Stomatology, Wuhan University, Wuhan, China; 5 Department of Endodontics, School & Hospital of Stomatology, Wuhan University, Wuhan, China; 6 Department of Pediatric Dentistry, University of Alabama at Birmingham, Birmingham, Alabama, United States of America; University of Toronto, CANADA

## Abstract

Knowledge of the polymicrobial etiology of root caries is limited. To conduct a comprehensive research study on root caries, we utilized 454-pyrosequencing of 16S rRNA gene libraries and quantitative PCR to compare supra-gingival bacterial communities from healthy sites and carious sites of 21 patients with root caries (Patient-controls and Patient-cases) and the sites of 21 healthy individuals (Healthy-controls) from two nursing homes. Healthy-controls and Patient-cases showed no significant differences in terms of biomass, species richness, and species diversity. However, as for beta diversity based on either community membership metric (unweighted UniFrac) or community structure metric (weighted UniFrac), Healthy-controls and Patient-cases were clearly distinguished from each other, appearing more variable in the community membership and structure in root caries microbiome but relatively conserved in the health microbiome. The Patient-controls group was at an intermediate stage between Healthy-controls and Patient-cases, but was more inclined to the former. Demonstrated in both relative abundance and prevalence of species in health and root caries, *Propionibacterium acidifaciens*, *Streptococcus mutans*, *Olsenella profusa*, *Prevotella multisaccharivorax*, and *Lactobacillus crispatus* were found to be most associated with root caries, whereas *Delftia acidovorans*, *Bacteroidetes[G-2] sp.*, *Lachnospiraceae[G-3] sp.*, and *Prevotella intermedia* are most associated with health. Our study provides a basis for further elucidating the microbial etiology of root caries in the elderly.

## Introduction

Root caries is a significant oral public health problem among humans due to improvements in health care, longer life expectancy, and increasing demand to maintain oral health [1,2]. As the name implies, root caries occurs at the root cementum or dentine, and is caused by a supra-gingival microbial biofilm. The microbiological nature of dental plaque associated with root caries differs from that of coronal caries, although it is still technically regarded as supra-gingival plaque [3]. The etiology of root caries is influenced by several factors, among which microbiological factors are critical [3,4]. However, thorough understanding of the effect of microbiological factors remains elusive because of our limited knowledge about their complex ecosystem.

Earlier studies based on cultivation suggested that filamentous bacteria, such as *Actinomyces* species, are involved in root caries etiology [5]. Several reports have demonstrated that *Streptococcus mutans* alone or combined with *Lactobacillus spp*. is detected more frequently in plaque overlying carious surfaces compared with healthy root surfaces [6]. Preza *et al*. used culture-independent molecular techniques, and indicated that putative etiological agents of root caries include *Streptococcus mutans*, *Lactobacilli*, *Actinomyces, Atopobium, Olsenella, Pseudoramibacter, Propionibacterium*, and *Selenomonas* [2]. Unfortunately, other studies have failed to determine specific species in the etiology of root caries [7–9]. Furthermore, previous studies did not provide a comprehensive view of bacterial communities and total diversity that are present in the supra-gingival environment.

With advancements in sequencing technologies, comprehensive studies to elucidate the differences between health and disease in complex communities have become possible. 454-Pyrosequencing of 16S rRNA genes, which opened a new era in microbial ecology, has allowed extensive sequencing of microbial populations in a high-throughput and cost-effective manner. It also provided the ability to comprehensively study bacterial community structures at the species level. To broaden our understanding of bacterial ecology on root caries, in the present study, using the abovementioned technique and qPCR, individuals from two nursing homes were investigated to compare supra-gingival plaque microbiomes obtained from both healthy sites and carious sites of patients with root caries (Patient-controls and Patient-cases) and healthy individuals (Healthy-controls).

## Materials and Methods

### Ethics Statement

All participants provided their written informed consent for this institutionally approved study. Approval for this study was obtained from both the Human Ethics Research committee of the Hospital of Stomatology Wuhan University and BGI Institutional Review Board.

### Participants

Subjects with root caries and healthy controls were recruited from two nursing homes in Wuhan (China). All of the subjects were examined orally prior to sampling. Definition and diagnosis of root caries were based on the criteria of the World Health Organization [10]. All subjects were required to have at least 20 existing natural teeth left, and have not used systemic antibiotics or an antibacterial mouth rinse for the past 3 months. For the root caries group, the subjects were required to exhibit at least one or more caries on their root surfaces. Healthy controls were required to have no caries either in the crown or root surfaces.

### Sample collection and preparation

All procedures were performed by a single calibrated examiner (X.H.). All subjects were asked to avoid eating or drinking 1 h before the sampling. For the Healthy-controls group and the Patient-controls group (the healthy root surfaces of patients with root caries), supra-gingival plaque were collected from multiple healthy root surfaces per subject (at least one site from each of the four quadrants) and pooled. For the Patient-cases group, plaque samples were collected from each root caries site and pooled. 21 individual samples from each group were immediately suspended in 300 μl reduced transport fluid (RTF) buffer, packed in coolers with cold packs supplemented with ice, and transported to the laboratory within 3 h, where they were frozen at −80°C until further analysis. Total bacterial genomic DNA was extracted from each sample using the Epicentre method (Epicentre, Madison, WI, USA) according to published protocols [11].

### Quantification of total load for specific taxa and total bacterial load

Total bacterial load was determined via qPCR using universal primers and *Streptococcus mutans* ATCC 700610 genomic DNA as standard. The total loads of *Lactobacillus*, *Bifidobacterium*, and *Streptococcus mutans* were respectively determined using genus-specific or species-specific primers.

### Preparation of 16S rRNA gene amplicon libraries and sequencing

First, PCR amplification targeting the V3–V5 region of 16S rRNA gene was individually performed by using fusion primers composed of 454 FLX Titanium sequencing primers (primer A, 5ʹ-CCATCTCATCCCTGCGTGTCTCCGACTCAG-3ʹ and primer B, 5ʹ-CCTATCCCCTGTGTGCCTTGGCAGTCTCAG-3ʹ), a unique 10 nt barcode, and SSU rRNA primers (338F, 5ʹ-ACTCCTACGGGAGGCAGCAG-3ʹ and 907R, 5ʹ-CCGTCAATTCMTTTGAGTTT-3ʹ). The cycling protocol was used as follows: 3 min initial denaturation at 94°C; 30 cycles of denaturation at 94°C for 30 s, annealing at 50°C for 30 s, elongation at 72°C for 45 s; and final extension at 72°C for 7 min. After PCR amplification, the amplicons were purified using AMPure beads. Then, emulsion PCR and sequencing were performed according to the manufacturer’s recommendations [12]. All of the sequences and associated metadata were deposited to the NCBI Sequence Read Archive under the accession number SRP032358.

### Sequence processing and analysis

All raw sequences were assigned to corresponding samples by allowing 1 mismatch to the barcode and 2 mismatches to the reverse primer (907R) by using Mothur (v1.25) [13]. After denoising by PyroNoise algorithm [14], sequences with ambiguous base call or a homopolymer >8 nt, or with a length of <200 nt or >1000 nt were removed. The sequences were aligned using an NAST-based sequence aligner to a custom reference based on the SILVA alignment. The sequences not aligned to the anticipated region of the reference alignment were removed, whereas the rest were pre-clustered by merging sequence counts that were no more than 3 nt different from a more abundant sequence. Chimeric sequences were identified using UCHIME algorithm [15] before removal. To classify the sequences, BLAST searches were carried out against HOMD 16S rRNA gene database (v12; http://www.homd.org/) with E-value <10^–4^. Next, the sequences were clustered into operational taxonomic units (OTUs) at 3% distance cutoff using the average neighbor clustering algorithm. Alpha and beta diversities based on UniFrac metrics were computed using QIIME programs (v1.5) [16].

### Statistical analysis

Among the demographic and clinical characteristics of the study subjects, age and number of remaining teeth (NRT) were compared between the healthy group and the group with root caries using Student’s t-test. Gender and smoking status were each compared via Fisher’s exact test. Due to the rarefaction curves of all 63 samples that were too dense to be discerned, the samples from the same group were averaged. Thus, each group was represented by only one rarefaction curve of the average with error bars of standard deviation. Differences in bacterial loads between groups were tested via Wilcoxon rank-sum test. Afterward, bacterial loads were transformed using natural logarithm (ln) to make the boxplot. The relative abundances of each taxon were compared between groups via Wilcoxon rank-sum test, whereas the prevalences of each taxon were compared using Fisher’s exact test. For alpha and beta diversity, the differences between groups were evaluated via Wilcoxon rank-sum test. Unless otherwise noted, the significance testings stated above used an alpha value of 0.05.

## Results

Supra-gingival bacterial communities were sampled from 21 healthy controls and from healthy and carious sites of 21 patients with root caries. Demographic and clinical metric for the participants are depicted in Table 1. No significant differences were observed between the healthy and root caries group for gender, age, and smoking status.

**Table 1:** Demographic and clinical characteristics of the study subjects.

	Root caries (n = 21)	Health (n = 21)	p values
Age^a^ (mean ± SD)	75.4 ± 6.8	73.3 ± 6.6	0.32
Gender female^b^ (%)	42.9	42.9	1.00
NRT^a^ (mean ± SD)	23.3 ± 2.4	25.6 ± 2.4	0.04*
RCT (mean ± SD)	6.3 ± 3.4	0	
RDFS (mean ± SD)	7.5 ± 3.6	0	
Smoking^b^ (%)	9.5	9.5	1.00

SD = standard deviation of the mean.

NRT = number of remaining teeth.

RCT = root caries teeth.

RDFS = root decayed filled surfaces.

^a^ Student’s t test.

^b^ Fisher’s exact test.

* p<0.05

** p<0.01

Amplicons from V3-V5 hypervariable regions of 16S gene were sequenced. After processing, our dataset included 144,408 sequences (ranging from 1,759 to 3,601 sequences per sample) with an average sequence length of 395 nt. By BLAST against HOMD, 11 phyla, 112 genera, and 227 species were detected.

### Verification of results

**Fig 1:**
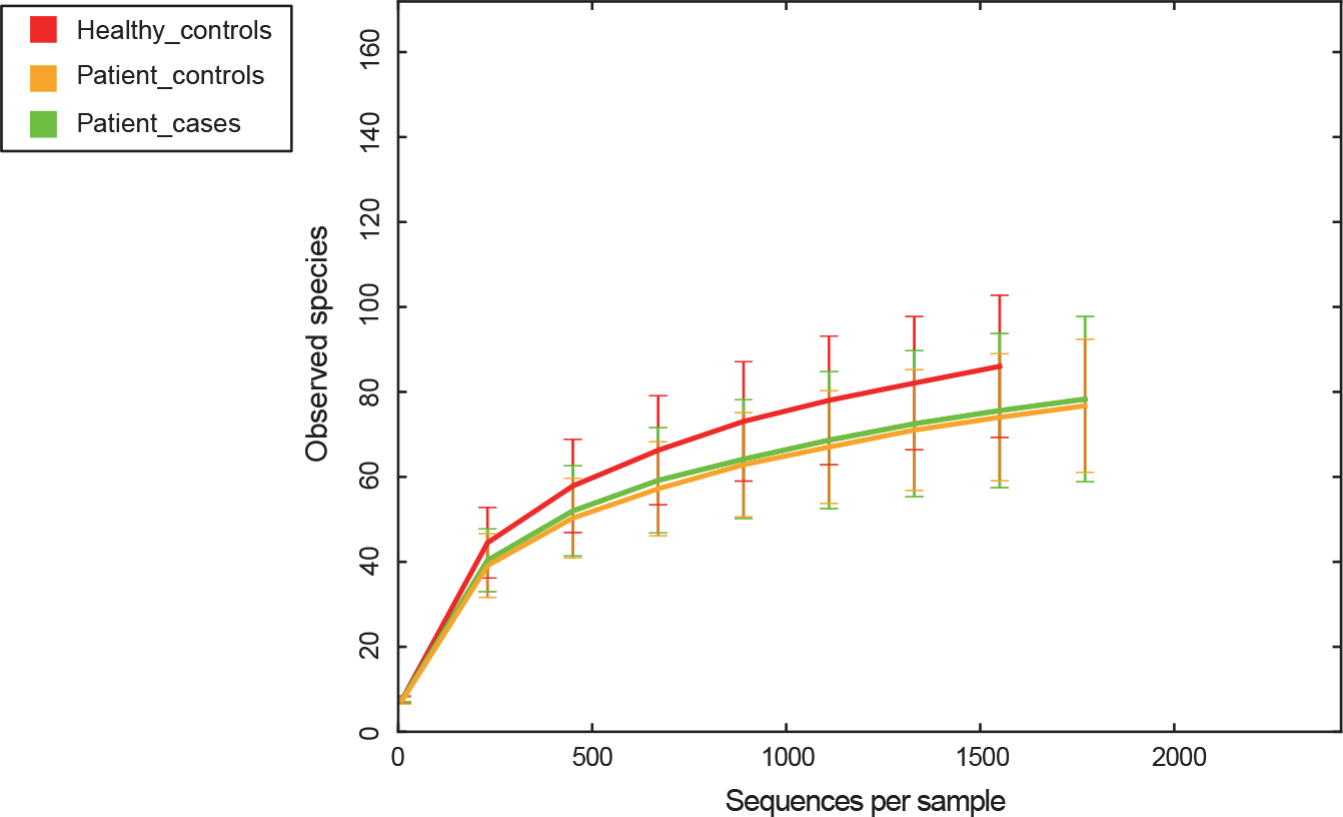
Rarefaction curves for evaluation of species coverage in our study. The *x* axis indicates the number of sequences per sample. The *y* axis indicates the average number of OTUs (0.03 distance threshold) per sample in each group. The error bars represent standard deviation.

To evaluate the species coverage identified by our sequencing effort, rarefaction analysis was performed (Fig. 1). Although the rarefaction curves did not achieve the even stage, the terminal slopes of them were rather low, suggesting that the sequencing detected the majority of the species. The undetected ones may be due to their quite low abundance in samples and deeper sequencing is required.

**Fig 2:**
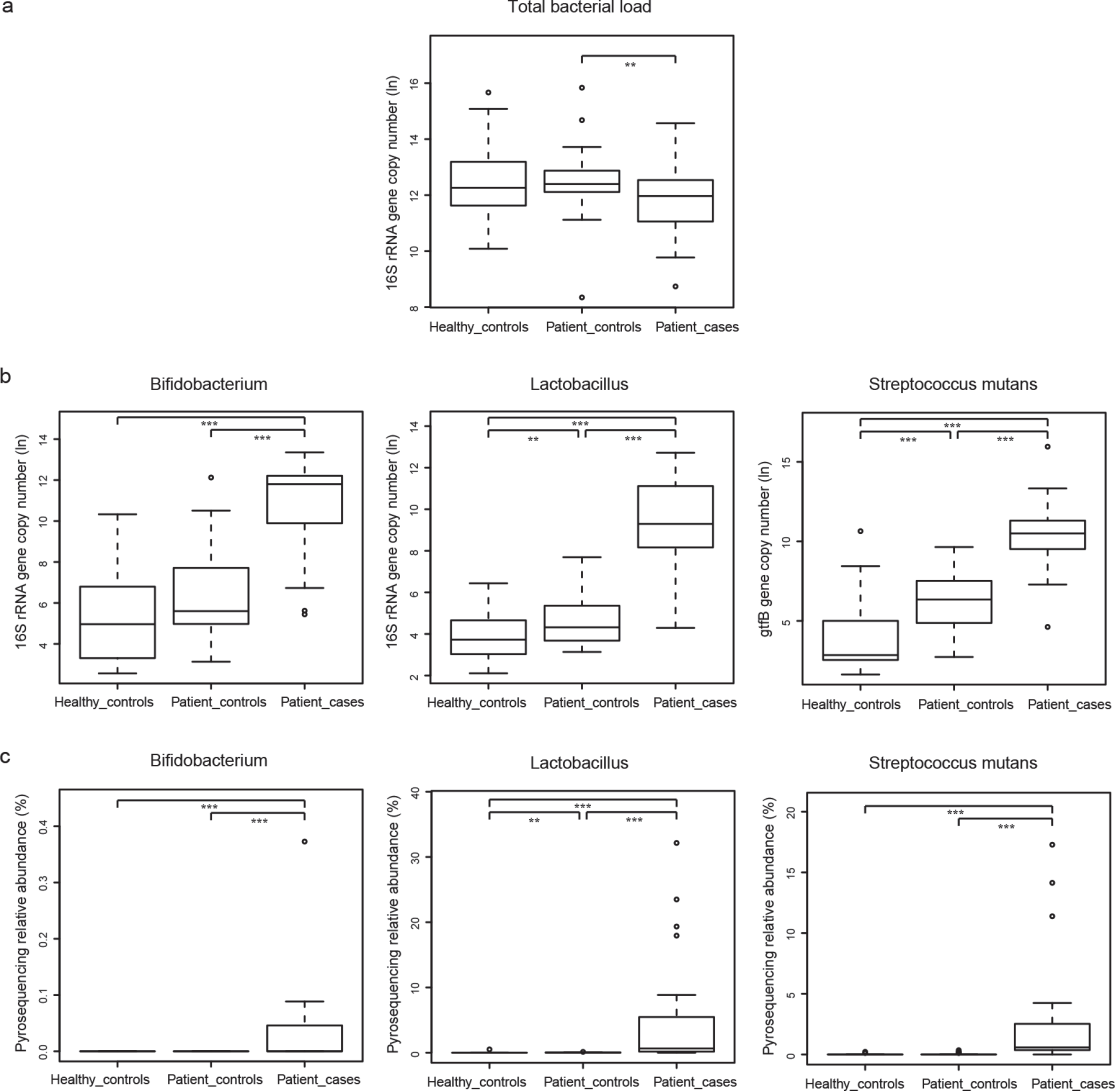
Boxplots for comparison of bacterial loads by qPCR and bacterial relative abundances by 454 pyrosequencing. (a) Boxplot for total bacterial load. (b) Boxplots for the total loads of genera *Bifidobacterium*, *Lactobacillus*, and species *Streptococcus mutans*. (c) Boxplots for the pyrosequencing relative abundances of the same taxa as (b). Gene copy number shown above was transformed by natural logarithm (ln) and relative abundance was transformed by percent (%). * denotes P < 0.1; ** denotes P < 0.05; *** denotes P < 0.01.

We utilized qPCR to quantify total bacterial load and total load of specific taxa in our samples. Restricted by the available DNA amount of samples, only three taxa were selected, including two genera of *Bifidobacterium*, *Lactobacillus* and one species of *Streptococcus mutans*. As seen in Fig. 2A, the difference in the total bacterial load was significant only between Patient-controls and Patient-cases, suggesting that in patients with root caries, the carious sites show higher biomass than the normal sites. Healthy-controls and Patient-cases showed no significant difference in total bacterial load, so we compared the three specific taxa’s loads (Fig. 2B) with their pyrosequencing relative abundances (Fig. 2C) under these two conditions. The qPCR load and pyrosequencing relative abundances of each taxon revealed similar distributions.

### Phylogenetic tree provided a global view of supra-gingival microbiome at the species level

**Fig 3:**
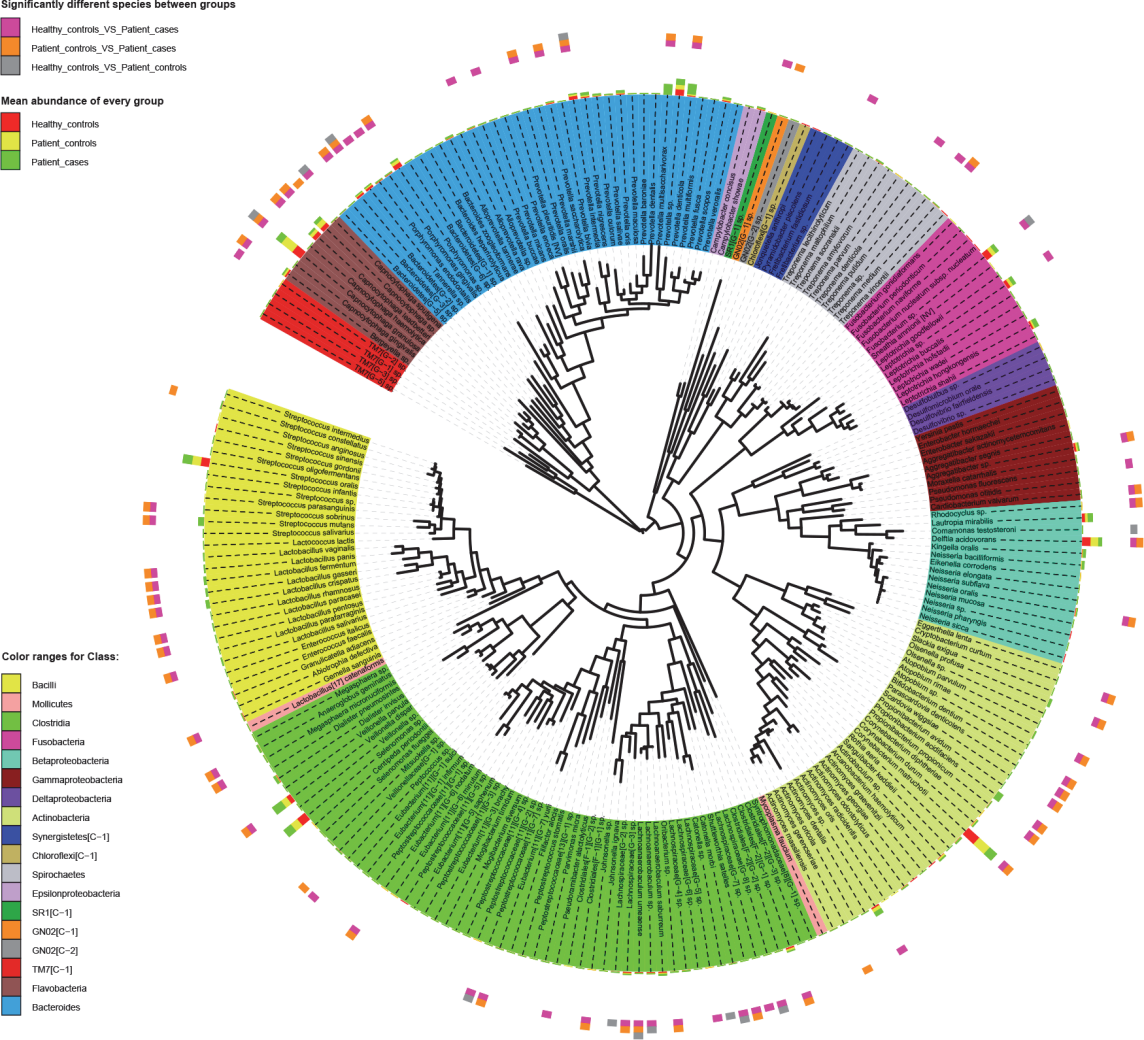
Maximum likelihood phylogenetic tree at the species level. Inner loop displays species with color ranges for the class level (see color key at the bottom left); middle loop indicates mean relative abundance in each group (see color key at the top left); outer loop indicates significantly different species detected between groups (see color key at the top left). An online tool of iTOL was used to construct this tree.

Fig. 3 shows the phylogenetic tree which included all the species detected by 454 sequencing, with their relative abundance in each group (Healthy-controls, Patient-controls, or Patient-cases) and the significant differences between groups. Most species (70) were significantly different between Healthy-controls and Patient-cases, followed by species (53) between Patient-controls and Patient-cases and species (13) between Healthy-controls and Patient-controls. We also found that 10 of 13 of the significant species between Healthy-controls and Patient-controls were also detected significantly between Healthy-controls and Patient-cases, suggesting that Patient-controls may be in a transitional stage to Patient-cases.

### Alpha diversity showed no significant difference between health and root caries, whereas beta diversity showed significant difference

**Fig 4:**
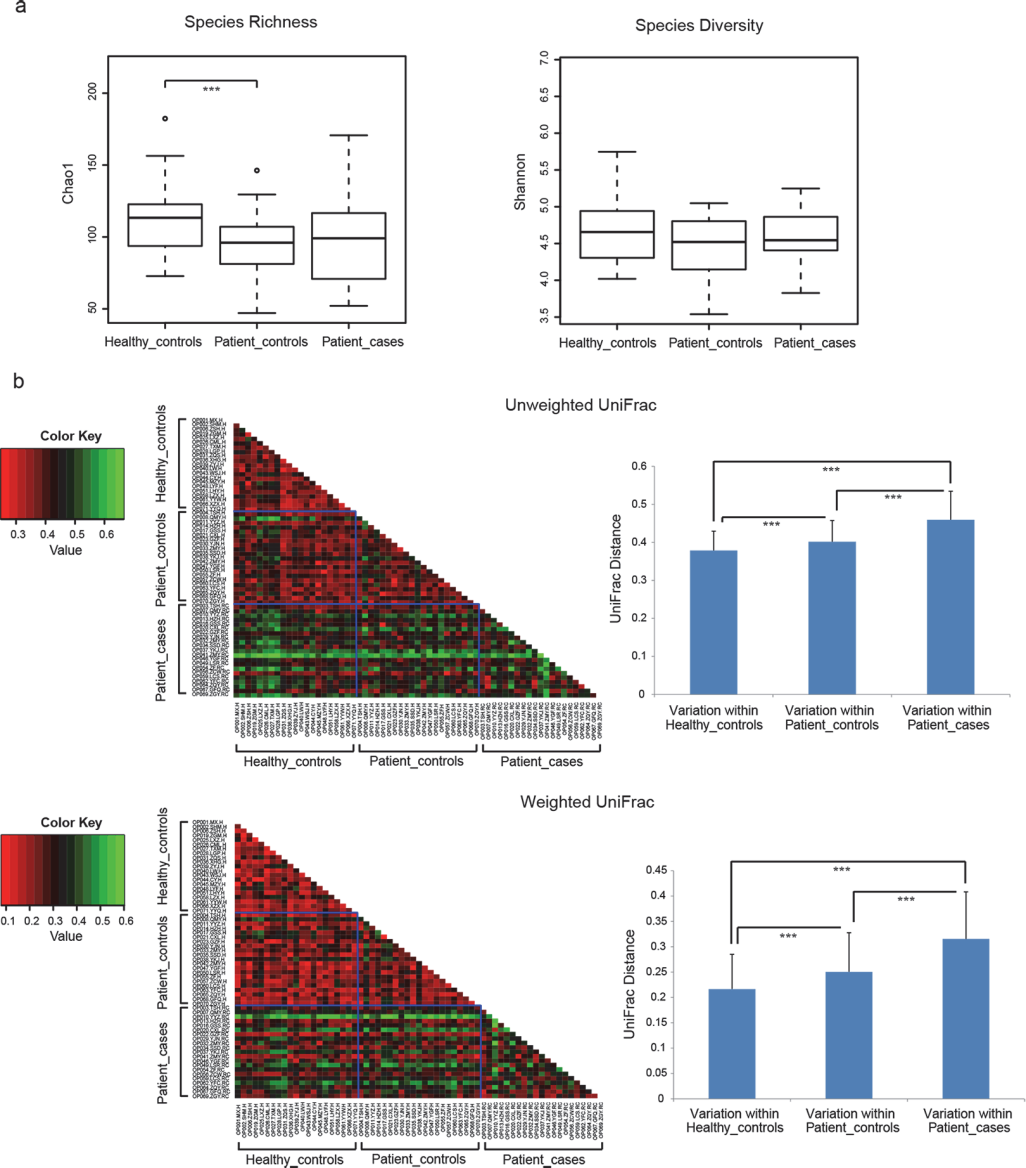
Alpha diversity and beta diversity. (a) Boxplots for alpha diversity metrics of Chao1 (species richness) and Shannon Diversity Index (species diversity). (b) Beta diversity metrics of unweighted UniFrac (community membership) and weighted UniFrac (community structure). The color in the heatmaps indicates the UniFrac distance between each pair of samples (see color key at the left). * denotes P < 0.1; ** denotes P < 0.05; *** denotes P < 0.01.

Fig. 4A (alpha diversity) shows that both Chao1 (species richness) and Shannon Diversity Index (species diversity) revealed no significant difference between Patient-cases and Healthy-controls or Patient-controls. The significant difference was only found between Healthy-controls and Patient-controls in Chao1. This may be due to inter-individual variability with more species observed in the 21 healthy individuals than in the 21 patients with root caries (Fig. 1).

Contrary to alpha diversity, beta diversity (Fig. 4B) showed variations between root caries and health. Based on either community membership metric (unweighted UniFrac) or community structure metric (weighted UniFrac), the variation within Patient-cases was significantly higher than that within Healthy-controls and that within Patient-controls. This suggests that community membership and structure of root caries microbiome were far more variable, whereas those of health microbiome were relatively conserved. Moreover, the variation within Patient-controls was intermediate between the other two conditions. This supports the previous view that Patient-controls may be in a transitional stage to Patient-cases. However, Patient-controls microbiome was more similar to Healthy-controls than Patient-cases (heatmaps in Fig. 4B).

### Distributions of significant phyla, genera, and species on abundance and prevalence displayed concrete bacteria discrepancy between health and root caries

**Fig 5:**
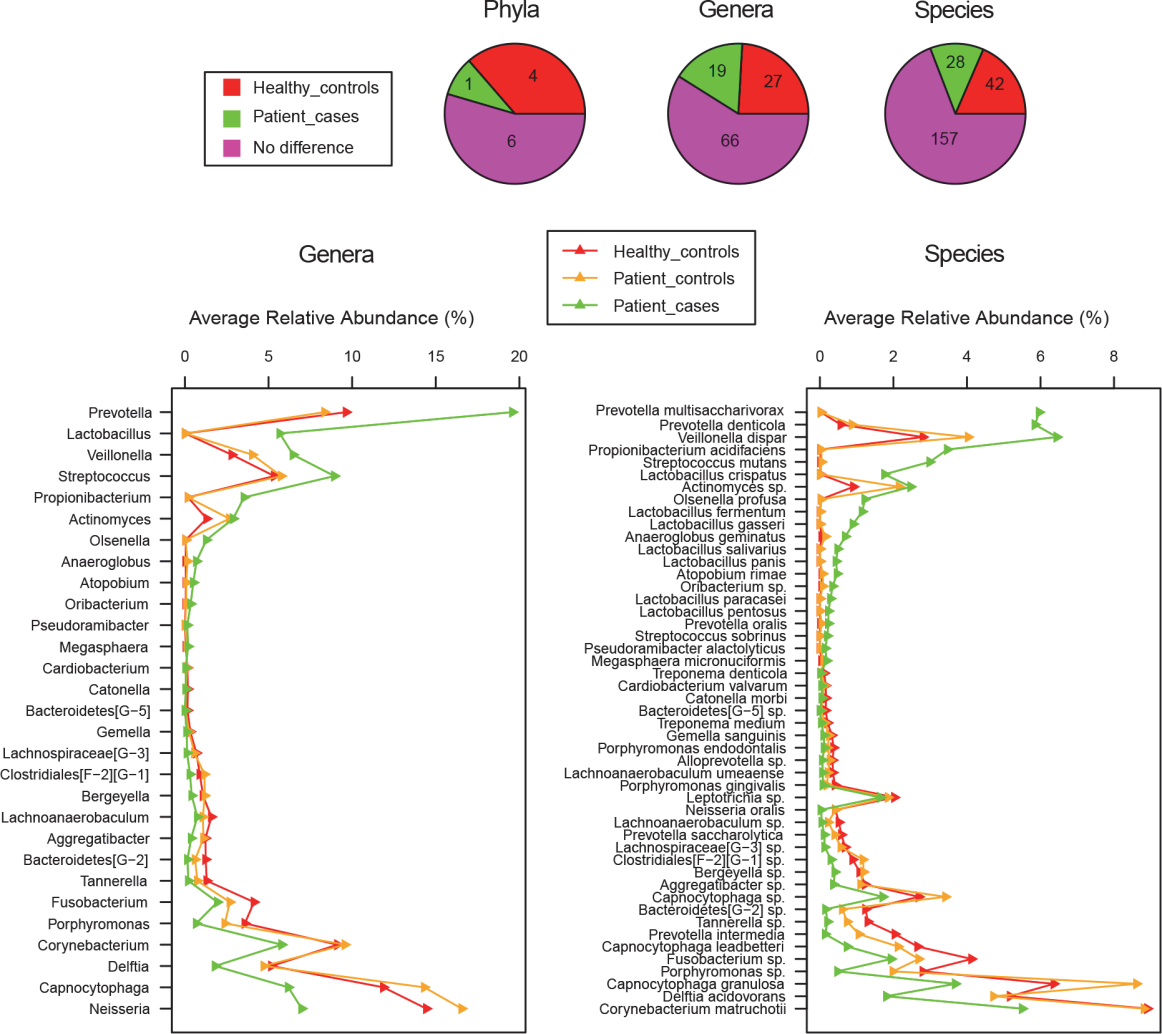
Distributions of abundance-based significant phyla, genera and species between health and root caries. Pie charts display the counts distribution of significantly different taxa. Line charts sort significantly different genera and species in the order of magnitude of abundance change (≥0.1%) between Healthy_controls and Patient_cases.

Fig. 5 reveals the phyla, genera, and species with significantly different relative abundances in health and root caries. As seen in the pie charts, 1 phylum, 19 genera, and 28 species were highly associated with disease, whereas 4 phyla, 27 genera, and 42 species were associated with health. The line charts sorted significant genera and species in the order of magnitude of abundance change between root caries and health, thus the most divergent genera and species were highlighted and they were more likely to be root caries pathogens or probiotics.

**Fig 6:**
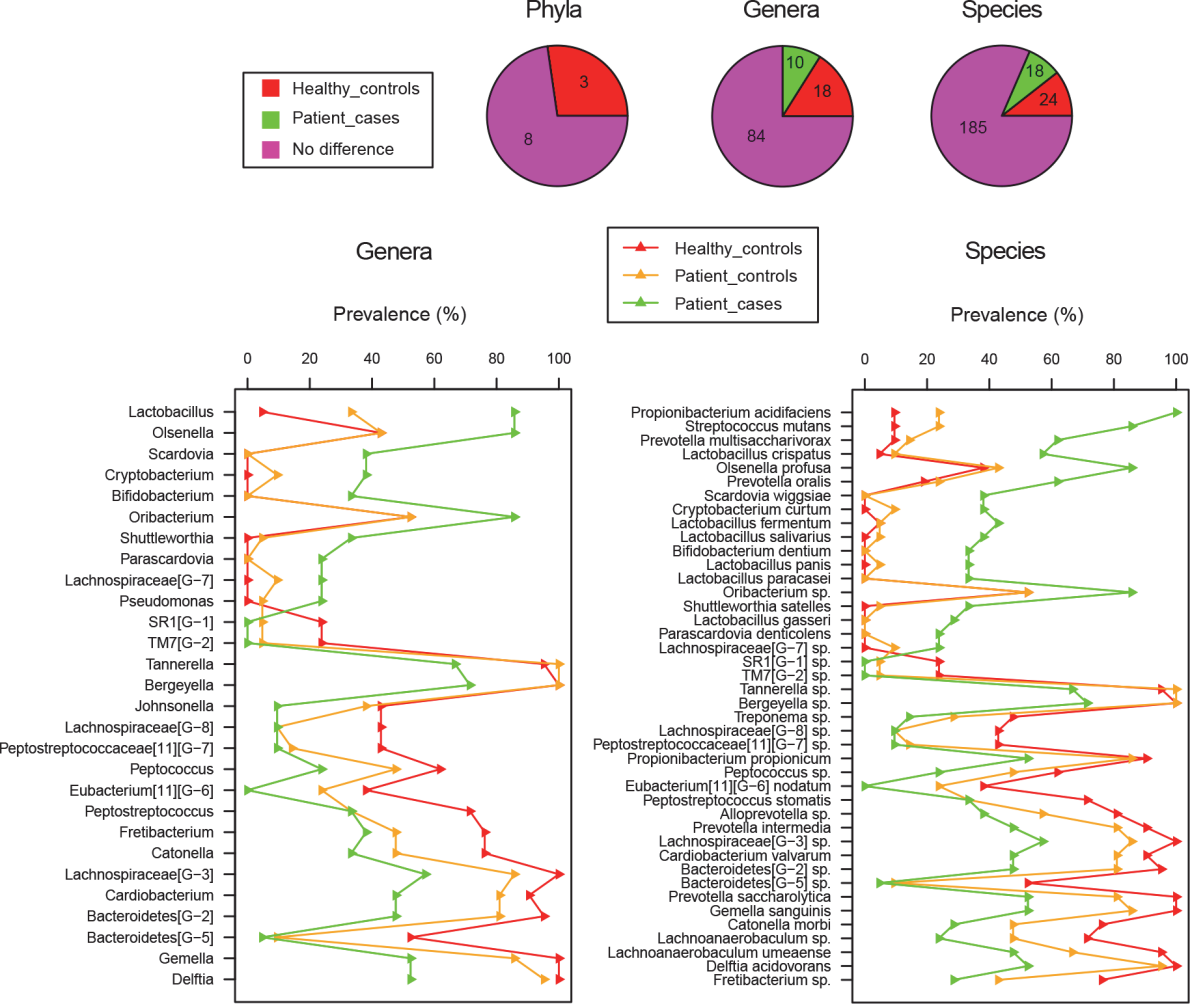
Distributions of prevalence-based significant phyla, genera and species between health and root caries. Pie charts display the counts distribution of significantly different taxa. Line charts sort significantly different genera and species in the order of magnitude of prevalence change between Healthy_controls and Patient_cases.

Furthermore, the phyla, genera, and species with significantly different frequencies of detection in health and root caries are shown in Fig. 6. A total of 0 phyla, 10 genera, and 18 species were associated with disease, whereas 3 phyla, 18 genera, and 24 species were associated with health. The line charts are similar to those in Fig. 5 but the significant genera and species were sorted by magnitude of prevalence change.

Thus, as indicated by the large magnitude of both abundance change (≥0.5%, Fig. 5) and prevalence change (≥30%, Fig. 6), the species *Propionibacterium acidifaciens*, *Streptococcus mutans*, *Prevotella multisaccharivorax*, *Lactobacillus crispatus*, *Olsenella profusa*, *Lactobacillus fermentum*, and the genera *Lactobacillus*, *Olsenella* are more likely to be root caries pathogens, whereas the species *Delftia acidovorans*, *Bacteroidetes[G−2] sp*., *Lachnospiraceae[G-3] sp*., *Prevotella intermedia*, and the genera *Delftia*, *Bacteroidetes[G−2]*, *Lachnospiraceae[G-3]* are more likely to be probiotics.

Furthermore, compared with Patient-controls, as seen in the line charts of Figs. 5 and 6, although a few genera and species distributed similarly to Patient-cases, the majority resembled Healthy-controls.

### Clustering presented the relationship and health status among samples

**Fig 7:**
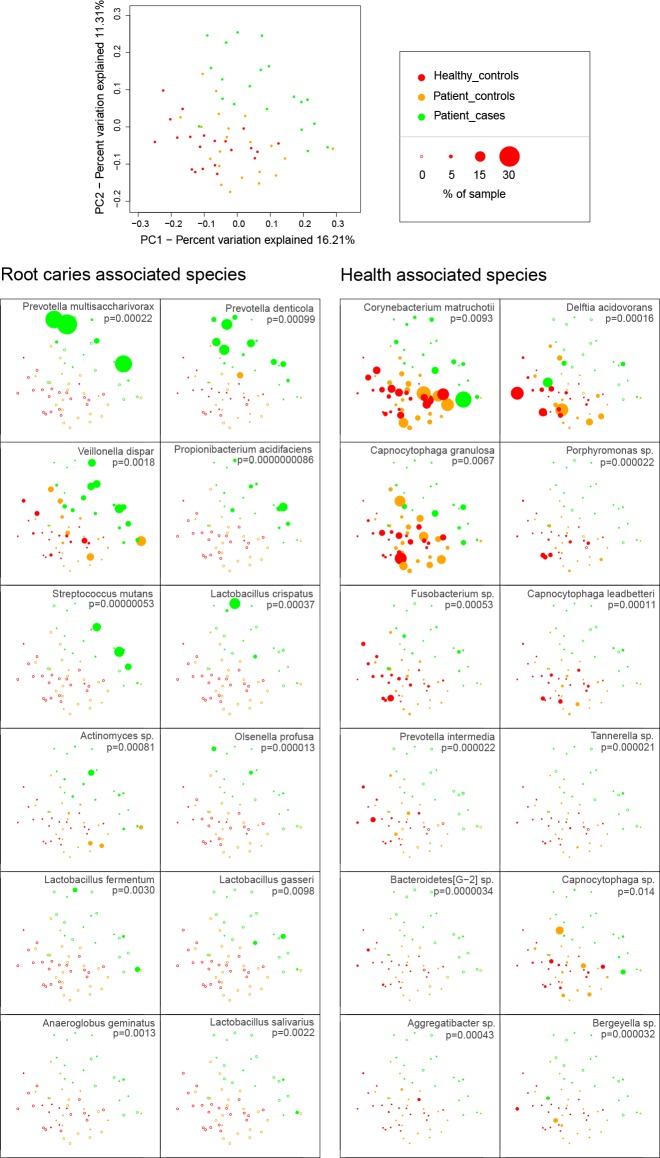
Samples clustering by PCoA. All PCoA plots were first constructed on the basis of unweighted UniFrac distance (see the panel at the top left). Then the points in PCoA plots were weighted by the relative abundances of the top 12 root caries-associated species and top 12 health-associated species of Fig. 5.

All samples were clustered by principal coordinates analysis (PCoA) based on unweighted UniFrac distance (Fig. 7). The samples of Patient-cases were clearly distinguished from those of Healthy-controls and Patient-controls, whereas no clear distinction was observed between Healthy-controls and Patient-controls.

To show the health status among all samples, the top 12 root caries-associated species and top 12 health-associated species (Fig. 5) were selected, and their own relative abundances in every sample were used to weight the corresponding points in PCoA.

### Core microbiome in health and root caries

**Fig 8:**
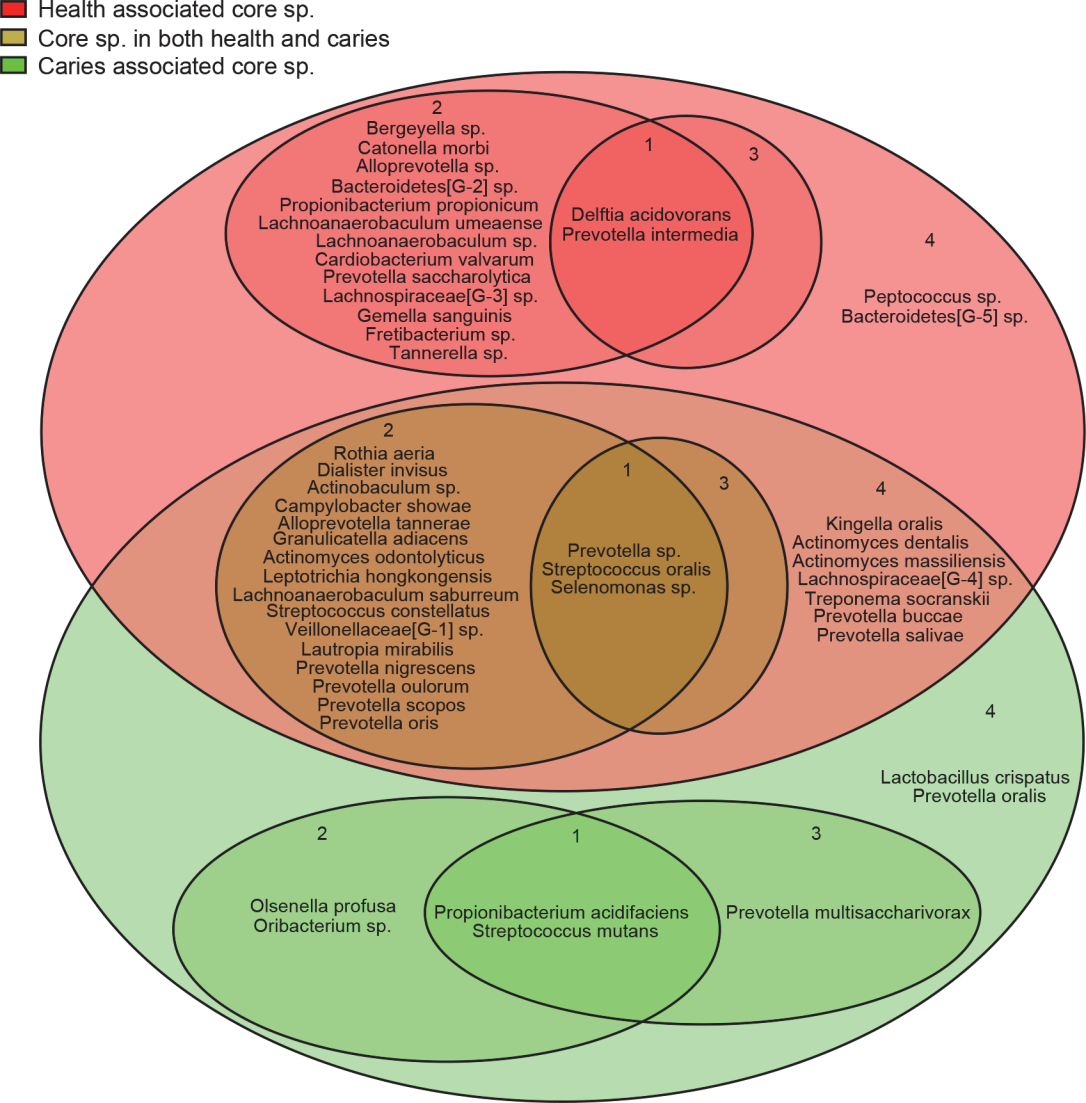
Core supra-gingival microbiome in root caries and health. Red field represents health-associated core species with significantly higher prevalence and relative abundance in Healthy_controls than in Patient_cases; green field represents root caries-associated core species with significantly higher prevalence and relative abundance in Patient_cases than in Healthy_controls; brown field represents core species in both health and root caries with no significantly different prevalence and relative abundance between the two groups. In each field, the prevalences of species were at least 1/2. Inner circles labeled 1 contain the species with high prevalence (prevalence ≥ 2/3) and high abundance (average relative abundance ≥ 2%); circles labeled 2 contain species with high prevalence (prevalence ≥ 2/3) but low abundance (average relative abundance < 2%); circles labeled 3 contain species with moderate prevalence (1/2 ≤ prevalence < 2/3) but high abundance (average relative abundance ≥ 2%); circles labeled 4 contain species with moderate prevalence (1/2 ≤ prevalence < 2/3) and low abundance (average relative abundance < 2%).

In this study, core microbiome was defined as the species which were detected in most individuals. Fig. 8 shows the health-associated core species (red field), root caries-associated core species (green field), and the core species in both health and root caries (brown field). Up to 26 species were contained in the brown field, indicating a steady foundation of microbiome in the supra-gingival environment regardless of being under health condition or root caries condition. The three species *Prevotella sp*., *Selenomonas sp*., and *Streptococcus oralis* in the inner circle of the brown field may play most important roles in forming the supra-gingival environment. Furthermore, the number of species (7) in the green field was quite less than that (17) in the red field. This result suggests that the microbiome composition in root caries was more variable than that in health. Considering such variability in root caries, the seven root caries-associated core species, especially *Propionibacterium acidifaciens* and *Streptococcus mutans*, are pivotal in the etiology of root caries. The seven species were chosen for the candidate cariogenic core microbiome. The two species *Delftia acidovorans* and *Prevotella intermedia* in the inner circle of the red field are crucial factors in maintaining oral health.

## Discussion

Root caries has a polymicrobial etiology and is emerging as a significant problem in humans. However, research on the bacterial community of root caries is difficult due to limitations in technology. The 454 pyrosequencing of 16S rRNA gene has made it possible to comprehensively elucidate the differences between disease and health at the species level. Using 454 pyrosequencing and qPCR, this study showed dependable and extensive results on supra-gingival microbiome structure and diversity and their discrepancy between root caries and health, thus expanding existing knowledge on putative pathogens and probiotics.

The consistency between 454 pyrosequencing and qPCR for some specific genera and species (Fig. 2) supports our results. In terms of biomass (Fig. 2A), species richness, and species diversity (Fig. 4A), Healthy-controls and Patient-cases showed no significant differences. This finding is different from that in periodontitis, in which dysbiosis is associated with increases in bacterial alpha diversity and biomass [17].

Based on either community membership metric (unweighted UniFrac) or community structure metric (weighted UniFrac), beta diversities (Fig. 4B) in Healthy-controls and Patient-cases were distinguishable from each other. More variable community membership and structure were observed in root caries microbiome, whereas relatively conserved ones were found in health microbiome. This finding agrees with Yang *et al*. who found similar results in the saliva of crown caries using 16S pyrosequencing method [18]. The discrepancies between Healthy-controls and Patient-cases were also exhibited by PCoA (Fig. 7).

In addition, Patient-controls was supposed to be in a transitional stage from health to root caries but was more inclined to health (Figs. 3, 4B, 5, and 6). This finding suggests that in patients with root caries, noticeable conversion in the microbiome composition of carious location was observed, but only a weak interference occurred in the normal neighborhood. Nevertheless, normal sites may have pathogens and should still be well maintained.

In this study, we investigated not only the abundant and prevalent species in health and root caries (Fig. 8), but also the change of taxa’ abundance and prevalence between health and root caries (Figs. 5 and 6). Through investigating the abundant and prevalent species in health and root caries, a steady foundation of microbiome in the supra-gingival environment was found regardless of being under health condition or root caries condition. *Prevotella sp*., *Selenomonas sp*., and *Streptococcus oralis* may have the most important functions in forming the supra-gingival environment. Although related metabolic studies about these species are deficient, *Streptococcus oralis* has been reported to produce neuraminidase, an IgA protease facilitated in biofilms such as dental plaque. Our data also confirm that *Prevotella sp*. and *Selenomonas sp*. are common in supra-gingival plaque, as revealed in a previous study using 16s clone. Moreover, 7 root caries-associated core species, especially *Propionibacterium acidifaciens* and *Streptococcus mutans*, and 17 health-associated core species, especially *Delftia acidovorans* and *Prevotella intermedia*, were shown in our result. Through investigating the change of taxa’ abundance and prevalence between health and root caries (Figs. 5 and 6), the species *Propionibacterium acidifaciens*, *Streptococcus mutans*, *Prevotella multisaccharivorax*, *Lactobacillus crispatus*, *Olsenella profusa*, *Lactobacillus fermentum*, and the genera *Lactobacillus*, *Olsenella* showed large magnitude of both relative abundance increase and prevalence increase from health to root caries, whereas the species *Delftia acidovorans*, *Bacteroidetes[G-2] sp*., *Lachnospiraceae[G-3] sp*., *Prevotella intermedia*, and the genera *Delftia*, *Bacteroidetes[G-2]*, *Lachnospiraceae[G-3]* exhibited large increase of magnitude from root caries to health.

Based on the abundant and prevalent species and their changes between health and root caries, *Propionibacterium acidifaciens*, *Streptococcus mutans*, *Olsenella profusa*, *Prevotella multisaccharivorax*, and *Lactobacillus crispatus* are considered to be root caries pathogens, whereas *Delftia acidovorans*, *Bacteroidetes[G-2] sp*., *Lachnospiraceae[G-3] sp*., and *Prevotella intermedia* are probiotics. Notably, *Prevotella* species were highlighted on both sides. As shown in previous studies, some *Prevotella* species are implicated in endodontics [19], and caries-active and healthy host populations carry different arrays of *Prevotella* species [18]. In addition, among the putative root caries pathogens, *Streptococcus mutans* has been extensively studied [20,21], but its specific role in root caries has not yet been proven. The other putative pathogens and probiotics have been minimally studied, and thus, warrant more attention.

In terms of prevalence (Fig. 6), among the 18 root caries-associated species, 10 species have zero prevalence in Healthy-controls, indicating that more than half of the species were not detected in health possibly due to their absence or their extremely low relative abundance. However, for the 24 health-associated species, only three were not detected in root caries. This finding suggests that root caries-associated species are more likely to obey the “Specific Plaque Hypothesis”, which indicates that caries result from the activity of specific microorganisms [21,22]. *Scardovia wiggsiae*, *Cryptobacterium curtum*, *Lactobacillus salivarius*, *Bifidobacterium dentium*, *Lactobacillus panis*, *Lactobacillus paracasei*, *Shuttleworthia satelles*, *Lactobacillus gasseri*, *Parascardovia denticolens*, and *Lachnospiraceae [G-7] sp*. are the 10 species that were detected only in root caries. The advent or the revival of these members may be the original factor that affected the foundation of healthy supra-gingival microbiome and ultimately the environment of root caries come into being. Health-associated species, the majority of which were also detected in root caries, seems inclined to the “Ecological Plaque Hypothesis” [23–25], which regards caries as a consequence of a populational balance shift of resident microbes, rather than a corollary of the disappearance of health-associated species.

In summary，we comprehensively examined the structure and diversity of supra-gingival microbiome in health and root caries of nursing home residents with similar demographic and clinic characteristics. Our study highlighted a list of putative root caries pathogens and probiotics from multiple aspects. Among the species, some of the results obtained were consistent with previous studies. However, these species warrant more attention. Apart from the highlighted ones, a large number of root caries-associated and health-associated taxa were extended. All these taxa can be utilized for further verifications and can provide clues for identifying the relationship between root caries and microbes. In addition, root caries influences the supra-gingival microbiome regionally, showing a remarkable microbiome conversion on carious locations but only a weak interference on its normal neighborhood. Both the “Specific Plaque Hypothesis” and the “Ecological Plaque Hypothesis” can partially elucidate our result. Future studies that examine individual sites over time as root caries progresses and at broader populations are required for better understanding the microbial pathogenesis of root caries in the elderly.
